# Metabolic Dysfunction-Associated Steatotic Liver Disease and Atrial Fibrillation Recurrence After Catheter Ablation: A Systematic Review and Meta-Analysis

**DOI:** 10.31083/RCM54276

**Published:** 2026-09-18

**Authors:** Panagiotis Theofilis, Paschalis Karakasis, Panayotis K. Vlachakis, Konstantinos Pamporis, Aikaterini Vordoni, Evangelos Oikonomou, Kyriakos Dimitriadis, Gerasimos Siasos, Konstantinos Tsioufis, Dimitris Tousoulis

**Affiliations:** ^1^Department of Cardiology, “Hippokration” General Hospital of Athens, 11527 Athens, Greece; ^2^Department of Cardiology, Ippokrateio General Hospital of Thessaloniki, 54642 Thessaloniki, Greece; ^3^Department of Cardiology, Sotiria General Hospital, 11527 Athens, Greece

**Keywords:** steatotic liver disease, liver fibrosis, atrial fibrillation, catheter ablation

## Abstract

**Background:**

Catheter ablation (CA) is an established rhythm-control strategy for atrial fibrillation (AF); however, AF recurrence remains common and is influenced by the underlying cardiometabolic substrate, including metabolic dysfunction-associated steatotic liver disease (MASLD). This study aimed to evaluate the association of MASLD and MASLD-related fibrosis risk with AF recurrence following CA.

**Methods:**

We conducted a systematic review and random-effects meta-analysis of observational studies reporting AF recurrence after CA, stratified by MASLD status and liver fibrosis risk, as assessed using the fibrosis-4 (FIB-4) index and the non-alcoholic fatty liver disease (NAFLD) fibrosis score (NFS). Eligible studies included adults undergoing CA for AF with recurrence outcomes reported and stratified by MASLD or fibrosis risk categories.

**Results:**

A total of 160 records were identified, and seven studies were included in the qualitative synthesis. Six studies, including 2921 patients, contributed to the primary meta-analysis comparing MASLD versus no MASLD. An additional fibrosis-only study contributed to fibrosis-stratified analyses. The meta-analysis of six studies (MASLD: 894; non-MASLD: 2027) demonstrated that MASLD was associated with a significantly higher risk of AF recurrence after CA (risk ratio (RR) 1.77, 95% confidence interval (CI) 1.43–2.18; *p* < 0.001; *I^2^* = 64.1%). In a separate meta-analysis of five studies reporting adjusted estimates, MASLD remained significantly associated with recurrence (hazard ratio (HR) 2.13, 95% CI 1.49–3.04; *p* < 0.001; *I^2^* = 70.5%). In fibrosis-stratified analyses, Intermediate/High FIB-4 (RR 2.03, 95% CI 1.27–3.26; *p* = 0.003; *I^2^* = 71.3%) and Intermediate/High NFS (RR 1.65, 95% CI 1.12–2.43; *p* = 0.01; *I^2^* = 49.9%) were associated with a higher risk of AF recurrence compared with low scores.

**Conclusion:**

MASLD is associated with a significantly higher risk of AF recurrence after CA. Fibrosis risk stratification using FIB-4 and NFS also shows a significant association between Intermediate/High fibrosis and increased recurrence rates.

**PROSPERO Registration:**

CRD420261335405, https://www.crd.york.ac.uk/PROSPERO/view/CRD420261335405.

## 1. Introduction

Atrial fibrillation (AF) is the most common sustained cardiac arrhythmia worldwide and is associated with substantial morbidity, including stroke, heart failure, and impaired quality of life [1,2,3]. Catheter ablation (CA) has become an established rhythm-control strategy for symptomatic patients and is widely used to improve arrhythmia control when antiarrhythmic drug (AAD) therapy is ineffective or poorly tolerated [4]. Despite advances in ablation techniques and patient selection, AF recurrence after the procedure remains common, highlighting the importance of identifying associated factors [5].

Metabolic dysfunction-associated steatotic liver disease (MASLD) is a common manifestation of cardiometabolic dysfunction and is closely linked to obesity, insulin resistance, hypertension, and diabetes [6]. Beyond hepatic involvement, MASLD has been increasingly recognized as a systemic condition associated with cardiovascular disease and AF [7]. Emerging observational studies have suggested that MASLD may be associated with a higher risk of AF recurrence following CA. In addition, the presence of liver fibrosis may identify patients at increased risk of recurrence. However, the available evidence remains limited. Therefore, the aim of the present study was to assess the association between MASLD and AF recurrence after CA and to explore whether MASLD-related fibrosis risk is an additional related factor.

## 2. Methods

The Preferred Reporting Items for Systematic Reviews and Meta-Analyses (PRISMA) 2020 guidelines were used in the preparation of this systematic review and meta-analysis (**Supplementary Table 1**) [8], which was preregistered in PROSPERO (ID: CRD420261335405).

### 2.1 Search Strategy

We performed a literature search in the PubMed, Scopus, and Web of Science databases from inception to 4 January 2026, without language restrictions. An additional search was performed before the first draft of the manuscript on 10 April 2026. Terms relevant to AF, CA, and MASLD were included, with the detailed queries shown in the **Supplementary Table 2**. Additionally, backward and forward citation tracking was performed using the citationchaser R package [9], and reference lists of included studies were manually screened. To enhance the breadth of the search, additional sources were explored, including manual searches of clinicaltrials.gov, the Epistemonikos database, and Google Scholar. Conference abstracts, preprints, and gray-literature records were considered eligible only if they provided sufficient data for outcome extraction or effect-size calculation.

### 2.2 Eligibility Criteria and Outcomes

Studies were eligible if they included adults (≥18 years) with AF who underwent CA and reported post-ablation AF recurrence according to MASLD status or MASLD-related fibrosis risk (e.g., fibrosis-4 (FIB-4) or non-alcoholic fatty liver disease (NAFLD) fibrosis score [NFS]) with follow-up beyond the blanking period. We included observational cohort or case-control studies reporting sufficient data to calculate effect estimates for AF recurrence in patients with and without MASLD or according to fibrosis risk categories.

We excluded non-original articles, as well as studies in which:

• Patients were not undergoing CA for AF (medical management only or other rhythm-control interventions).

• MASLD/NAFLD or liver fibrosis risk was not defined or stratified using recognized diagnostic criteria or validated non-invasive fibrosis scores.

• AF recurrence outcomes after CA were not reported or could not be extracted/calculated according to MASLD or fibrosis status.

• Outcomes were limited to procedural or in-hospital results without post-blanking follow-up assessment of AF recurrence.

• Duplicate or overlapping populations were reported (in which case the most complete or recent dataset was included).

### 2.3 Study Selection and Data Extraction

All records identified through the database search were imported into Rayyan (https://new.rayyan.ai/) for screening and selection. After automatic removal of duplicates, two reviewers (P.T., P.K.V.) independently screened titles and abstracts to identify studies evaluating the association between MASLD and AF recurrence following CA. Discrepancies were resolved via consensus or by consultation with a third reviewer.

The full text of studies deemed suitable was assessed on the basis of predefined eligibility criteria.

Extracted variables included:

• Study characteristics: first author, publication year, study design (retrospective/prospective).

• Population characteristics: total number of participants, AF subtype (paroxysmal or persistent), mean age, male sex distribution, cardiometabolic comorbidities (e.g., diabetes mellitus (DM), hypertension), body mass index (BMI), AAD use, and follow-up duration.

• Procedural characteristics: CA modality (radiofrequency (RF) or cryoballoon ablation), CA strategy (pulmonary vein isolation (PVI) alone or PVI with additional lesion sets such as cavotricuspid isthmus or linear lesions).

• Exposure definitions: diagnostic criteria used for MASLD and the use of non-invasive fibrosis scores, including the FIB-4 and NFS.

• Outcomes and their definition: AF recurrence and monitoring following CA.

### 2.4 Risk of Bias Assessment

The Newcastle-Ottawa Scale (NOS) for observational studies was employed for risk of bias assessment. For comparative cohort studies, all three NOS domains (Selection, Comparability, and Outcome) were applied, yielding a maximum score of 9 stars. Studies were then classified as low risk of bias (7–9 stars), moderate risk of bias (4–6 stars), or high risk of bias (≤3 stars) [10].

### 2.5 Statistical Analysis

All meta-analyses were conducted using the meta, metafor, and dmetar packages in R Studio (version 1.4.1106; Posit Software, PBC, Boston, MA, USA). For the primary MASLD versus non-MASLD comparison, unadjusted risk ratios (RRs) with 95% confidence intervals were calculated from study-level recurrence events and group denominators. Fibrosis-risk analyses using FIB-4 and NFS were also pooled as unadjusted RRs comparing Intermediate/High-risk versus low-risk categories. In a separate analysis, multivariable-adjusted hazard ratios (HRs) were pooled for studies reporting adjusted time-to-event estimates for the association between MASLD and AF recurrence. Adjusted HRs and unadjusted RRs were analyzed separately and were not combined in the same meta-analysis. Because of presumed clinical and methodological heterogeneity across studies due to differences in MASLD definitions, ablation techniques, and rhythm monitoring protocols, a random-effects model (DerSimonian–Laird method) was used as the pooling method. Statistical heterogeneity was assessed using the *I^2^* statistic, with values of approximately 25%, 50%, and 75% interpreted as low, moderate, and high heterogeneity, respectively. Forest plots were generated to visually display individual study estimates and pooled results.

Publication bias was assessed by visual inspection of funnel plots. Since fewer than 10 studies were available for each synthesis, formal evaluation of small-study effects and publication bias was considered unreliable, and no quantitative asymmetry testing was performed.

Sensitivity analyses were performed by sequentially omitting one study at a time (leave-one-out analysis) to evaluate the robustness of the pooled estimates and to determine whether any single study disproportionately influenced the overall results. To explore potential sources of between-study heterogeneity, prespecified subgroup analyses were performed for the primary MASLD versus non-MASLD comparison when at least two studies were available per subgroup. Studies were stratified according to MASLD definition, categorized as imaging-based versus fatty liver index/metabolic dysfunction-based definitions, and by follow-up duration, categorized as >14 months versus ≤14 months based on the approximate median follow-up across included studies. Subgroup analysis according to monitoring intensity was considered but not performed because monitoring protocols were heterogeneous, inconsistently reported, and not amenable to a reproducible study-level classification given the small number of included studies.

## 3. Results

### 3.1 Study Selection

The study selection process is documented in Fig. 1. A total of 160 records were initially identified through database searches. After removal of duplicates, 132 unique records remained for screening. Following title and abstract screening, 121 records were excluded for not meeting inclusion criteria. The full text of 11 reports was assessed for eligibility. Of these, 5 were further excluded. Before the first draft of the manuscript, an additional search was performed, which identified one more eligible study. Ultimately, 7 studies were considered suitable for data extraction and inclusion in the meta-analysis.

**Fig. 1.**
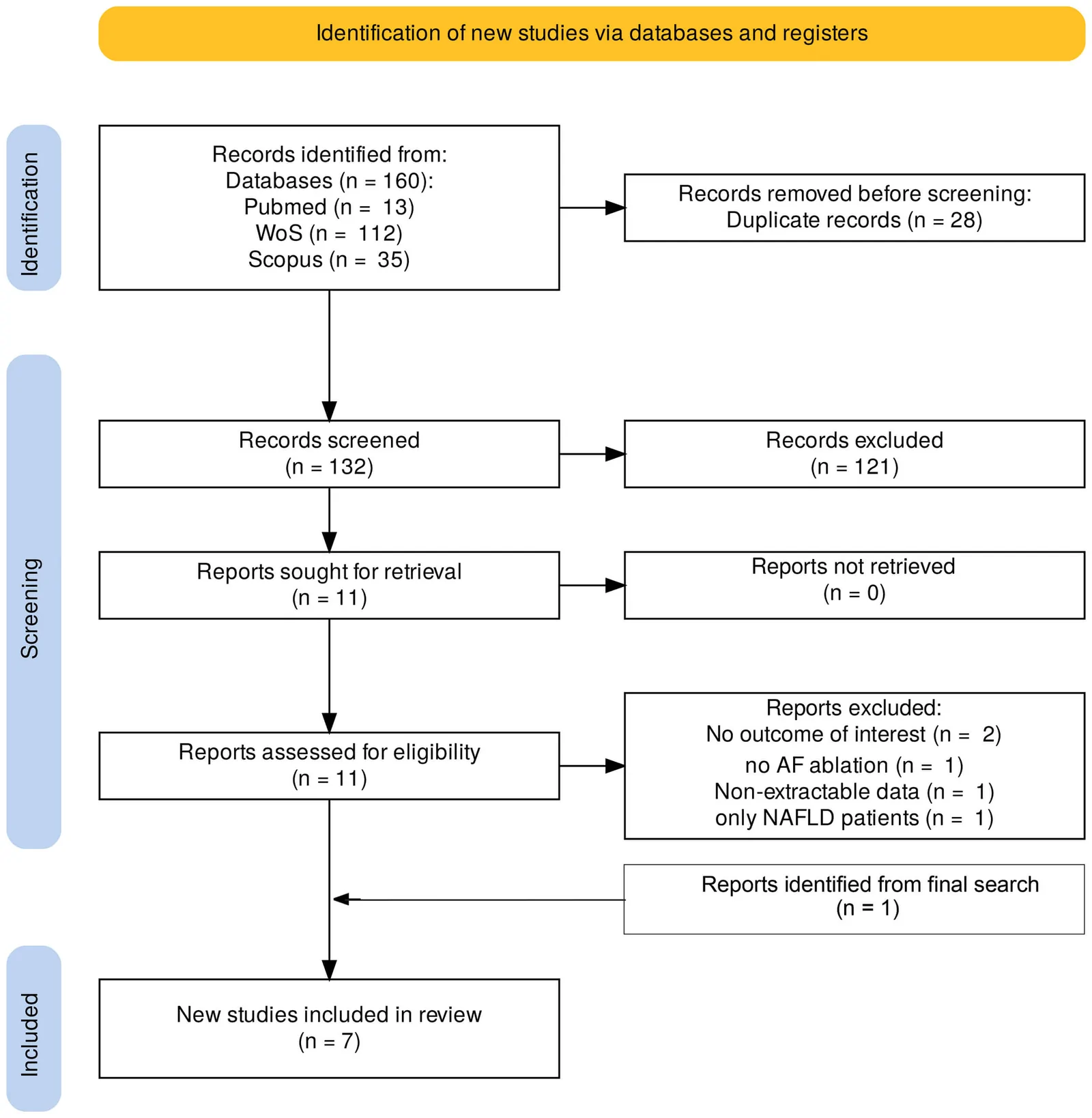
**PRISMA flowchart of the study selection process**. WoS, Web of Science; AF, atrial fibrillation; NAFLD, non-alcoholic fatty liver disease.

### 3.2 Qualitative Synthesis

Seven observational studies were included in the qualitative synthesis (Table 1, Ref. [11,12,13,14,15,16,17]), all published between 2020 and 2026 [11,12,13,14,15,16,17]. Six studies reported AF recurrence after catheter ablation according to MASLD status and were included in the primary MASLD versus non-MASLD meta-analysis. One additional study provided fibrosis-stratified data only and was included in the fibrosis-risk analyses. Most had a retrospective design, while two were prospective [11,17]. The sample sizes ranged from 106 to 1182. Overall study quality was moderate to high (**Supplementary Table 3**). Two studies scored 8/9 points [12,15], three studies 7/9 [14,16,17], and two studies 6/9 [11,13], indicating low to moderate risk of bias across the included cohorts. Most studies showed strong performance in the selection and outcome domains, with clearly defined AF recurrence and structured follow-up. Lower scores were mainly observed in the comparability domain, reflecting limited adjustment for potential confounders.

**Table 1.** **Main characteristics of the included studies**.

Study	Year	Study design	AF type	CA type	CA lesions	FU (months)	Fibrosis scoring
Takami et al. [15]	2026	Retrospective	Paroxysmal 65.2% Persistent 34.8%	RF 44.7% Cryo 55.3%	PVI + CTI	24.3	NFS FIB-4
Donnellan et al. [12]	2020	Retrospective	NR	RF 98% Cryo 2%	PVI 100% PW 40% Roof 18–16% Septum 23–20% CTI 17%	29.0 (17.0)	NFS
Wang et al. [16]	2025	Retrospective	Paroxysmal 53.9% Persistent 46.1%	RF	PVI only 79–83%	12.0	NR
Wang et al. [11]	2025	Prospective	Paroxysmal 61.1% Persistent 38.9%	Cryo	PVI only	14.0	NR
Wang et al. [14]	2022	Retrospective	Paroxysmal 59.7% Persistent 40.3%	RF	NR	12.0	NFS FIB-4
Decoin et al. [13]	2022	Retrospective	NR	RF	PVI only 79–83% PVI+lines 21–17%	13.9 (6.6, 28.9)	NFS
Xu et al. [17]	2026	Prospective	Paroxysmal 64.1% Persistent 35.9%	RF 72.6% Cryo 27.4%	NR	12.3 (1.3)	FIB-4

Follow-up values in parentheses -when available- include either standard deviation or interquartile range. CA, catheter ablation; NR, not reported; FU, follow-up; Cryo, cryoablation; RF, radiofrequency; PVI, pulmonary vein isolation; CTI, cavotricuspid isthmus; PW, posterior wall; FIB-4, fibrosis-4; NFS, NAFLD fibrosis score.

Across studies, both paroxysmal and persistent AF populations were represented, although the proportion of persistent AF varied considerably. All but one study [12] reported inclusion of patients undergoing first CA. Donnellan et al. [12] did not clearly specify whether all patients were undergoing first-time CA. RF ablation was the dominant strategy in most studies, whereas one study included exclusively cryoballoon ablation and another included both RF and cryoballoon approaches. PVI was the core lesion set across all studies, with some cohorts also including cavotricuspid isthmus ablation and additional linear lesions at operator discretion.

Detailed AAD protocols were not consistently reported across studies (**Supplementary Table 4**). Baseline AAD use was reported in four studies, ranging from 29.9% to 86.5% in MASLD patients and from 36.1% to 83.1% in non-MASLD patients where comparator data were available. Post-ablation AAD strategies also differed. Several studies prescribed or continued AADs during the 3-month blanking period, while subsequent discontinuation was generally recommended or left to physician-patient discretion. One study used an AF subtype-specific approach, with AADs prescribed for persistent AF under physician guidance but not routinely used in paroxysmal AF. AADs were then discontinued after the blanking period unless symptomatic arrhythmias recurred. In two studies, post-ablation AAD management was not reported.

There was also methodological variation in how MASLD was defined, as the included studies did not uniformly apply the contemporary definition (**Supplementary Table 5**). Four studies used imaging-based definitions, relying on ultrasonography, computed tomography (CT), histology, or combinations thereof after exclusion of secondary causes of hepatic steatosis (NAFLD-style definitions). In contrast, two studies applied metabolic dysfunction-based frameworks, using a Fatty Liver Index (FLI) threshold combined with cardiometabolic abnormalities [11,13]. Despite this nomenclature and diagnostic heterogeneity, all studies compared recurrence after CA according to the presence or absence of MASLD, and several also evaluated fibrosis risk using non-invasive scores, most commonly the FIB-4 and NFS.

Definitions of recurrence were relatively consistent across the included studies (**Supplementary Table 5**). Most defined recurrence as documented AF, atrial flutter, or atrial tachyarrhythmia lasting more than 30 seconds after a standard 3-month blanking period. Monitoring protocols, however, differed in intensity (**Supplementary Table 5**). Some studies used structured follow-up with scheduled clinic visits, serial 12-lead electrocardiograms (ECGs), and repeated Holter monitoring, whereas others additionally incorporated transtelephonic rhythm monitoring, implantable devices, or symptom-triggered testing.

Baseline characteristics also suggested that the MASLD groups carried a less favorable cardiometabolic profile (**Supplementary Table 6**). Where reported, patients with MASLD tended to have a higher burden of diabetes, hypertension, higher body mass index, and in some studies a greater prevalence of persistent AF.

Median follow-up duration ranged from 12 to 29 months. Across individual studies, the direction of effect was largely consistent, with MASLD associated with higher AF recurrence after CA. The fibrosis-focused studies further suggested that liver fibrosis severity may identify a higher-risk phenotype within patients with MASLD. Taken together, these data support the concept that fibrosis scores may reflect a more advanced systemic and atrial remodeling milieu, although the number of available studies remains limited.

### 3.3 Meta-analysis

#### 3.3.1 MASLD and AF Recurrence After CA

Six studies including 2921 patients (MASLD: 894, non-MASLD: 2027) reported AF recurrence after CA according to MASLD status. In the pooled random-effects analysis (Fig. 2), MASLD was associated with a significantly higher risk of AF recurrence compared with non- MASLD (RR 1.77, 95% confidence interval (CI) 1.43–2.18, *p* < 0.001). Substantial between-study heterogeneity was observed (*I^2^* = 64.1%, *τ^2^* = 0.0425, *p* = 0.02). A leave-one-out sensitivity analysis was conducted to assess the influence of individual studies on the pooled estimate of MASLD and AF recurrence (**Supplementary Fig. 1**). Sequential exclusion of each study did not alter the overall effect size, with pooled estimates ranging from RR 1.55 to RR 1.80, and all remaining statistically significant.

**Fig. 2.**
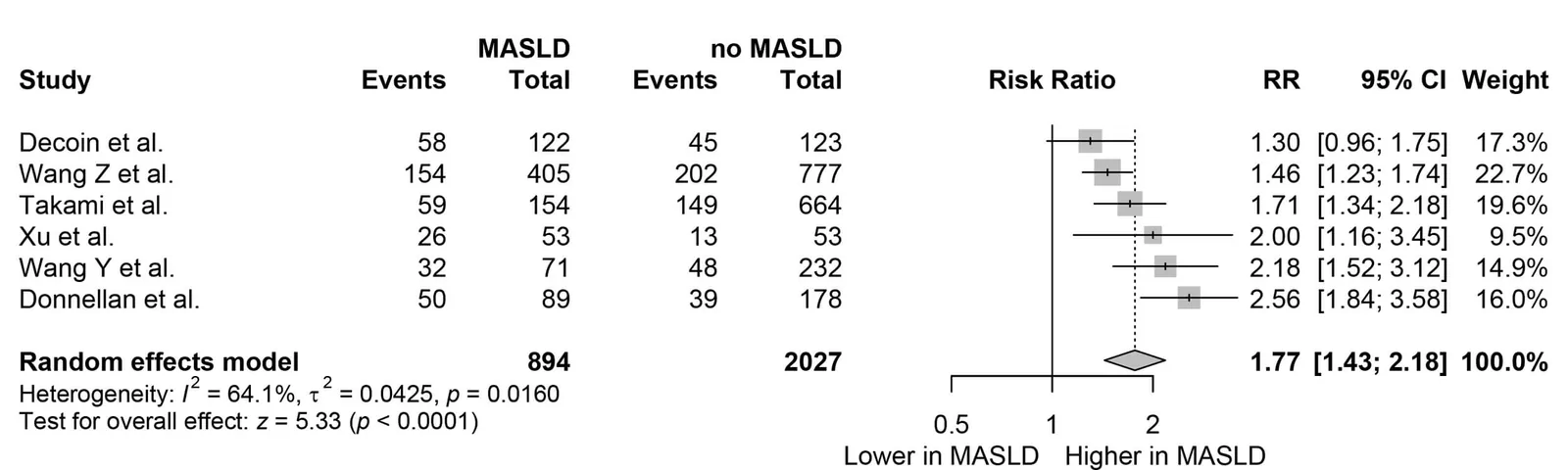
**Forest plot of AF recurrence after CA in patients with and without MASLD**. MASLD, metabolic dysfunction-associated steatotic liver disease; RR, risk ratio.

To account for potential confounding in observational studies, a separate meta-analysis was performed including studies reporting adjusted hazard ratios (HRs). In this analysis of five studies (Fig. 3), MASLD remained significantly associated with a higher risk of AF recurrence (HR 2.13, 95% CI 1.49–3.04, *p* < 0.001), with high heterogeneity (*I^2^* = 70.5%, *τ^2^* = 0.0967, *p* = 0.009). The list of confounders in each study is presented in **Supplementary Table 7**.

**Fig. 3.**
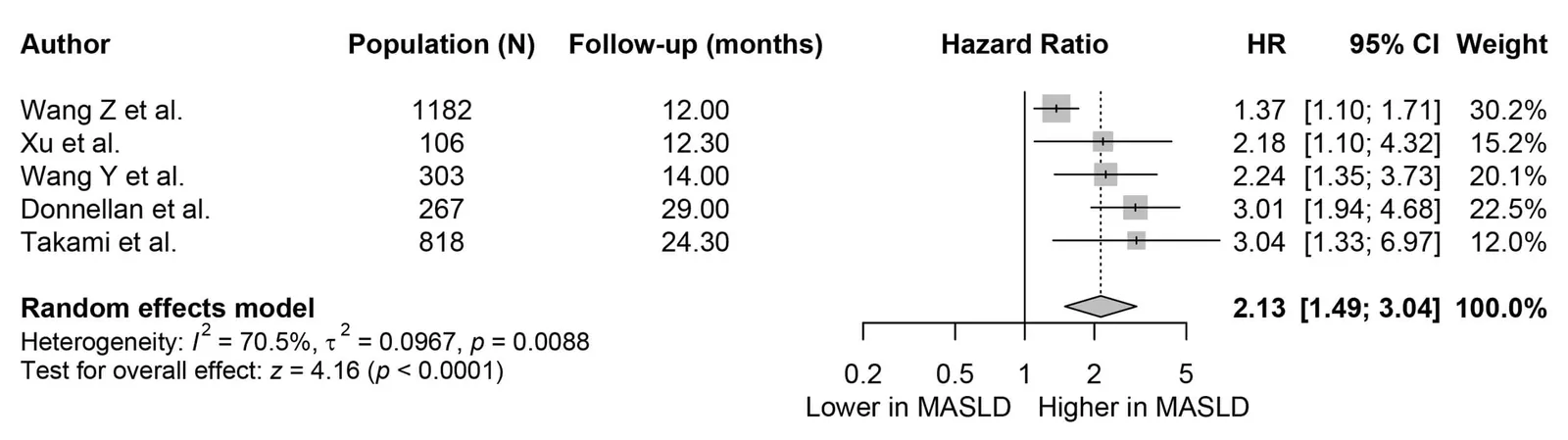
**Meta-analysis of adjusted hazard ratios for AF recurrence associated with MASLD**.

#### 3.3.2 Fibrosis Risk and AF Recurrence After CA

Fibrosis risk stratification was evaluated using FIB-4 and NFS. Four studies including 710 patients (Intermediate/High FIB-4: 429, low FIB-4: 281) were included in the FIB-4 analysis (Fig. 4A). Patients with Intermediate/High FIB-4 had a higher risk of AF recurrence, (RR 1.74, 95% CI 1.11–2.72, *p* = 0.003). Between-study heterogeneity was considerable (*I^2^* = 68.1%, *τ^2^* = 0.1034, *p* = 0.04).

**Fig. 4.**
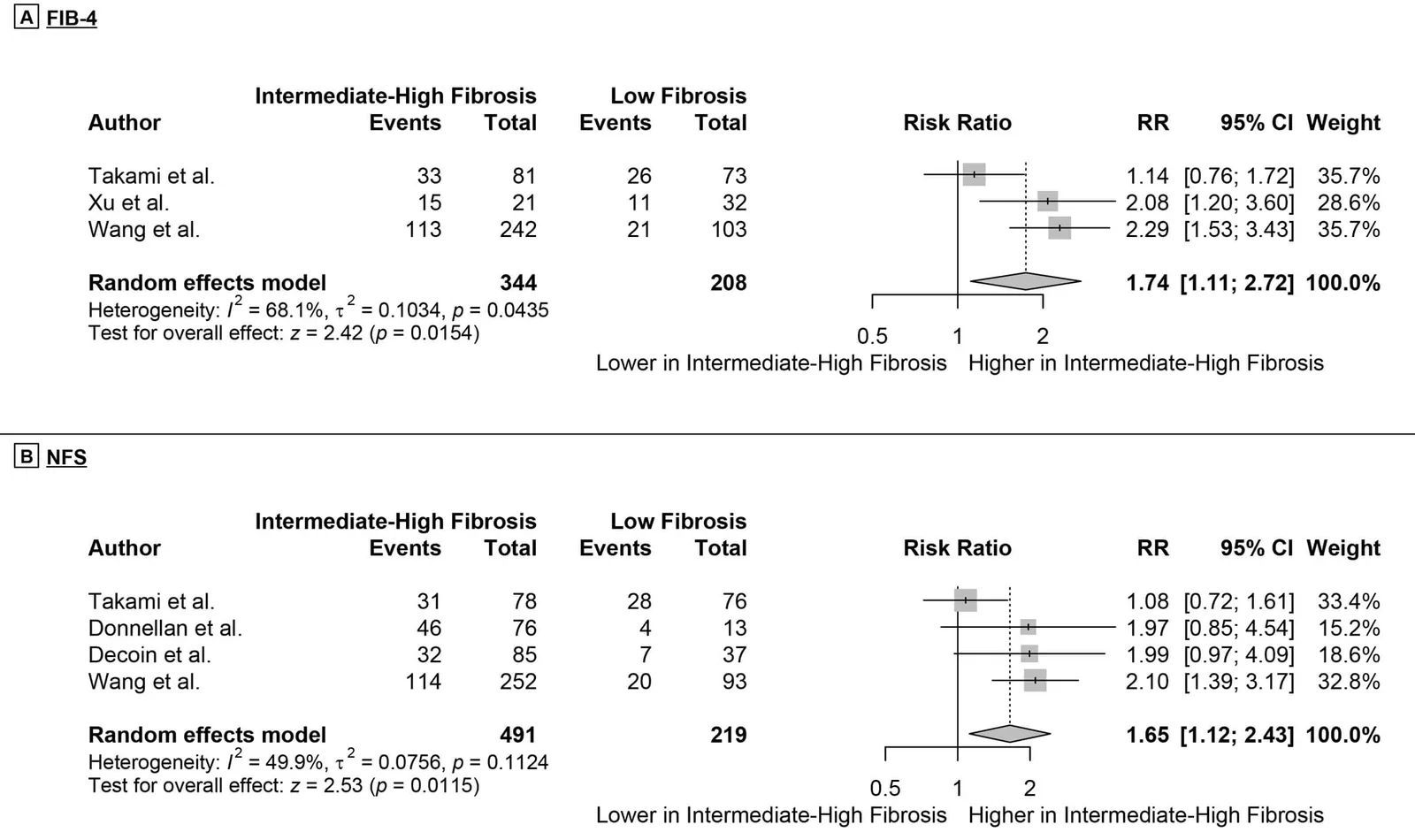
**Forest plot of AF recurrence after CA according to (A) FIB-4 and (B) NAFLD fibrosis score (NFS)**.

Four studies including 710 patients (Intermediate/High NFS: 491, low NFS: 219) evaluated fibrosis risk according to NFS (Fig. 4B). Intermediate/High NFS was associated with increased AF recurrence (RR 1.65, 95% CI 1.12–2.43, *p* = 0.01). Heterogeneity was moderate (*I^2^* = 49.9%, *τ^2^* = 0.0756, *p* = 0.11).

#### 3.3.3 Subgroup Analysis

Subgroup analyses were performed to explore potential sources of heterogeneity in the association between MASLD and AF recurrence after CA (**Supplementary Fig. 2**). Studies were stratified according to MASLD definition (imaging-based vs FLI-based) and follow-up duration (>14 months vs ≤14 months).

The pooled estimate remained directionally consistent across subgroups. For studies using imaging-based MASLD definitions, the pooled risk ratio for AF recurrence was RR 1.83 (95% CI 1.41–2.36), while studies using FLI-based definitions showed a similar effect (RR 1.67, 95% CI 1.00–2.76). There was no significant interaction between MASLD definition and AF recurrence risk (*p*-interaction = 0.75). Similarly, subgroup analysis according to follow-up duration showed comparable results. Studies with follow-up >14 months reported a pooled effect of RR 2.06 (95% CI 1.38–3.06), whereas studies with ≤14 months follow-up demonstrated RR 1.61 (95% CI 1.28–2.03). No significant subgroup interaction was observed (*p*-interaction = 0.30).

#### 3.3.4 Publication Bias

Publication bias was evaluated using contour-enhanced funnel plots for the MASLD, NFS, and FIB-4 analyses (**Supplementary Figs. 3–5**). Visual inspection of the funnel plots did not reveal evidence of substantial asymmetry, although interpretation is limited due to the small number of included studies in each analysis (<10). Consequently, formal statistical testing for funnel plot asymmetry was not performed.

## 4. Discussion

In this systematic review and meta-analysis, MASLD was associated with a significantly higher risk of AF recurrence after CA (Graphical Abstract). Importantly, this association remained significant when analyses were restricted to studies reporting adjusted estimates, suggesting that MASLD may represent an independent risk marker rather than purely reflecting the burden of accompanying cardiometabolic comorbidities. Analyses examining liver fibrosis risk using FIB-4 and NFS showed higher recurrence rates among patients with intermediate or high fibrosis risk. Overall, these findings suggest that MASLD identifies a higher-risk cardiometabolic phenotype among patients undergoing CA, rather than establishing a causal relationship between MASLD and AF recurrence.

Increasing evidence indicates that MASLD is part of a broader systemic cardiometabolic syndrome that may influence atrial structure and electrophysiology. Epidemiological studies have consistently shown that MASLD is associated with a higher incidence of AF and with markers of adverse cardiac remodeling [18,19,20]. The present findings extend this concept to the post-CA setting, suggesting that the metabolic and inflammatory milieu associated with MASLD may contribute to arrhythmia recurrence even after PVI [21,22]. Several pathophysiological mechanisms may explain this association. First, MASLD is characterized by chronic low-grade inflammation, oxidative stress, and activation of profibrotic signaling pathways [23]. These processes promote myocardial fibrosis and may facilitate atrial structural remodeling [24]. This interpretation is supported by recent work linking systemic redox imbalance to atrial structural remodeling. In a cross-sectional study of patients with AF, Özdemir Tutar et al. [25] reported that native thiol levels were modestly associated with echocardiographic indices of left atrial remodeling, although the authors emphasized that these findings were hypothesis-generating rather than definitive. This supports the broader concept that oxidative stress and inflammatory-metabolic pathways may contribute to atrial substrate vulnerability [25]. Second, MASLD frequently coexists with obesity, insulin resistance, hypertension, and diabetes, all of which are well-established risk factors for AF recurrence and are linked to atrial enlargement, fibrosis, and electrical remodeling [26]. The clustering of these cardiometabolic abnormalities may therefore contribute to a proarrhythmic atrial substrate that persists despite catheter-based rhythm control strategies. Analyses examining fibrosis risk showed a consistent direction of effect toward higher AF recurrence in patients with intermediate or high fibrosis scores. Fibrosis scores such as NFS and FIB-4 may capture a more advanced stage of metabolic disease and systemic fibrotic activity. Supporting this concept, previous work has demonstrated that patients with higher fibrosis scores exhibit more extensive atrial low-voltage [27].

Moderate-to-substantial heterogeneity was observed across several analyses. This likely reflects both clinical and methodological differences between the included studies. MASLD definitions varied, with some studies using CT, ultrasonography, imaging/histology, or American Association for the Study of Liver Diseases (AASLD)-based imaging/biopsy criteria after exclusion of secondary causes, whereas others used fatty liver index-based definitions combined with metabolic dysfunction criteria. Differences in recurrence ascertainment may have contributed to heterogeneity and potential detection bias. Although most studies defined recurrence as atrial tachyarrhythmia lasting >30 seconds after a 3-month blanking period, some definitions included only AF whereas others included AF, atrial flutter, or atrial tachycardia. Rhythm-monitoring protocols also varied substantially, ranging from scheduled ECG and Holter monitoring to transtelephonic rhythm transmitters, implantable device interrogation, event monitoring, symptom-triggered evaluation, and 3-day Holter monitoring. Studies using more intensive rhythm surveillance may detect more asymptomatic recurrences, which could influence recurrence rates and reduce comparability across cohorts. In addition, baseline cardiometabolic risk profiles differed across cohorts, with MASLD patients generally showing higher diabetes, hypertension, and BMI where reported. The distribution of persistent AF also varied across studies, which may reflect differences in atrial substrate severity and post-ablation recurrence risk. Finally, adjusted models included different covariate sets, including variable adjustment for BMI, diabetes, AF type, AF duration, left atrial size, biomarkers, and ablation modality. Therefore, the pooled estimates should be interpreted as average associations across heterogeneous observational cohorts rather than evidence of a uniform effect across all settings.

This study has several strengths. To our knowledge, this is the first meta-analysis specifically evaluating the relationship between MASLD and AF recurrence following CA. The analysis incorporated both unadjusted and adjusted estimates, allowing for a more robust assessment of the association while partially accounting for confounding factors inherent to observational research. Furthermore, we explored fibrosis risk using validated non-invasive scores, providing additional insight into the potential role of disease severity in arrhythmia recurrence.

## 5. Limitations

Several limitations should also be acknowledged. First, Embase was not searched separately. Therefore, eligible records indexed exclusively in Embase may have been missed. This limitation was mitigated by the use of multiple bibliographic databases and supplementary search approaches, including citation tracking and gray-literature searches. Furthermore, all included studies were observational, and therefore residual confounding cannot be excluded. The number of available studies was relatively small, particularly for fibrosis analyses, precluding formal evaluation of publication bias and performance of further subgroup analyses. It should also be noted that exposure definitions varied across studies, and most included studies did not use the contemporary MASLD nomenclature. Although prior evidence suggests substantial overlap between NAFLD and MASLD populations [28], cardiometabolic risk criteria were not uniformly required or reported in all included cohorts. Therefore, the pooled estimate combines related but non-identical steatotic liver disease phenotypes, and the findings should be interpreted as applying to MASLD or MASLD-equivalent steatotic liver disease. Additionally, rhythm monitoring protocols differed between studies, which could influence the detection of recurrence. Moreover, peri-procedural and post-ablation AAD protocols were not consistently reported across studies, precluding assessment of whether AAD continuation or discontinuation modified the association between MASLD and AF recurrence. Importantly, monitoring intensity differed across studies and may have introduced detection bias. Because rhythm monitoring protocols were heterogeneous and often combined scheduled ECG/Holter monitoring with symptom-triggered evaluation or device-based monitoring when available, a reliable subgroup analysis according to monitoring intensity was not feasible with the small number of included studies. Along those lines, although subgroup analyses by diagnostic approach showed directionally consistent findings, these analyses were exploratory and underpowered. A non-significant interaction *p*-value should therefore not be interpreted as evidence that all diagnostic definitions are identical. Finally, all included studies evaluated conventional RF or cryoballoon CA. Data on newer energy sources, such as pulsed field ablation, were not available in the current literature, and therefore the present findings may not extend to populations undergoing pulsed field ablation.

These findings have potential clinical implications, primarily for risk stratification and future research. MASLD is common among patients with AF and may identify a subgroup with a more adverse cardiometabolic and hepatic-risk profile before catheter ablation. Recognition of MASLD and fibrosis risk may therefore help clinicians contextualize recurrence risk alongside established factors such as AF subtype, obesity, diabetes, hypertension, left atrial size, and ablation strategy. However, the present meta-analysis is based on observational data and does not establish that MASLD directly causes recurrence or that treatment of MASLD reduces post-ablation arrhythmia recurrence. Future studies should aim to clarify the mechanistic links between MASLD and atrial remodeling and to determine whether targeted metabolic interventions, such as glucagon-like peptide 1 receptor agonists [29], can improve rhythm outcomes after CA. The potential reversibility of cardiometabolic cardiac remodeling is also supported by contemporary bariatric surgery data. In a prospective comparison of sleeve gastrectomy and Roux-en-Y gastric bypass, Maher et al. [30] observed significant improvements in metabolic, inflammatory, and echocardiographic parameters after bariatric surgery, with myocardial functional improvement associated with changes in HbA1c, C-reactive protein, and left ventricular mass rather than surgical procedure alone. Prospective multicenter studies using standardized definitions of MASLD and systematic rhythm monitoring will be essential to confirm these observations. In addition, integrating metabolic and hepatic parameters into existing AF risk prediction models may help refine patient selection and optimize long-term outcomes after ablation.

## 6. Conclusion

In this meta-analysis, MASLD was associated with a higher risk of AF recurrence following CA. These findings suggest that MASLD may identify patients with a more adverse cardiometabolic and hepatic risk profile who are at increased risk of recurrence. Future prospective studies using standardized MASLD definitions, systematic rhythm monitoring, and adequate adjustment for cardiometabolic confounders are needed to determine whether structured metabolic and hepatic-risk modification can reduce recurrence after catheter ablation.

## Data Availability

The datasets used and analyzed during the current study are available from the corresponding author on reasonable request.
