# Supplementary material for: Metabolic Dysfunction-Associated Steatotic Liver Disease and Atrial Fibrillation Recurrence After Catheter Ablation: A Systematic Review and Meta-Analysis
Source: Rev Cardiovasc Med. 2026 Sep 18;27(9):54276. doi: 10.31083/RCM54276 (PMC13653178; doi:10.31083/RCM54276)
Supplement: Supplementary file 1 [file 2153-8174-27-9-54276-s1.zip › Supplementary Material.docx]

**Supplementary Material**

Contents

[**Supplementary Table 1**. Preferred Reporting Items for Systematic Reviews and Meta-Analyses (PRISMA) checklist	1](#_Toc234067096)

[**Supplementary Table 2**. Search strategies for Pubmed, Scopus, and Web of Science	4](#_Toc234067097)

[**Supplementary Table 3.** Risk of bias assessment.	6](#_Toc234067098)

[**Supplementary Table 4.** Antiarrhythmic drug use in the included studies.	7](#_Toc234067099)

[**Supplementary Table 5.** Definitions of MASLD, AF recurrence and protocol for monitoring recurrence.	8](#_Toc234067100)

[**Supplementary Table 6.** Additional population characteristics of the included studies.	11](#_Toc234067101)

[**Supplementary Table 7.** Confounders adjusted for in multivariate models of the association between MASLD and AF recurrence after CA.	12](#_Toc234067102)

[**Supplementary Fig. 1.** Leave-one-out sensitivity analysis of the MASLD meta-analysis.	13](#_Toc234067103)

[**Supplementary Fig. 2.** Subgroup analysis of MASLD and AF recurrence after CA.	14](#_Toc234067104)

[**Supplementary Fig. 3.** Contour-enhanced funnel plot assessing publication bias for the MASLD meta-analysis.	15](#_Toc234067105)

[**Supplementary Fig. 4.** Contour-enhanced funnel plot assessing publication bias for the FIB4 meta-analysis.	16](#_Toc234067106)

[**Supplementary Fig. 5.** Contour-enhanced funnel plot assessing publication bias for the NFS meta-analysis.	17](#_Toc234067107)

# **Supplementary Table 1**. Preferred Reporting Items for Systematic Reviews and Meta-Analyses (PRISMA) checklist

| **Section and Topic** | **Item #** | **Checklist item** | **Location where item is reported** |
| --- | --- | --- | --- |
| **TITLE** | | |  |
| Title | 1 | Identify the report as a systematic review. | p. 1 |
| **ABSTRACT** | | |  |
| Abstract | 2 | See the PRISMA 2020 for Abstracts checklist. | p. 1 |
| **INTRODUCTION** | | |  |
| Rationale | 3 | Describe the rationale for the review in the context of existing knowledge. | p. 1 |
| Objectives | 4 | Provide an explicit statement of the objective(s) or question(s) the review addresses. | p. 1 |
| **METHODS** | | |  |
| Eligibility criteria | 5 | Specify the inclusion and exclusion criteria for the review and how studies were grouped for the syntheses. | p. 2 |
| Information sources | 6 | Specify all databases, registers, websites, organisations, reference lists and other sources searched or consulted to identify studies. Specify the date when each source was last searched or consulted. | p. 2 |
| Search strategy | 7 | Present the full search strategies for all databases, registers and websites, including any filters and limits used. | p. 2 / Supplementary Table 2 |
| Selection process | 8 | Specify the methods used to decide whether a study met the inclusion criteria of the review, including how many reviewers screened each record and each report retrieved, whether they worked independently, and if applicable, details of automation tools used in the process. | p. 2 |
| Data collection process | 9 | Specify the methods used to collect data from reports, including how many reviewers collected data from each report, whether they worked independently, any processes for obtaining or confirming data from study investigators, and if applicable, details of automation tools used in the process. | p. 2 |
| Data items | 10a | List and define all outcomes for which data were sought. Specify whether all results that were compatible with each outcome domain in each study were sought (e.g. for all measures, time points, analyses), and if not, the methods used to decide which results to collect. | p. 2 |
|  | 10b | List and define all other variables for which data were sought (e.g. participant and intervention characteristics, funding sources). Describe any assumptions made about any missing or unclear information. | p. 2 |
| Study risk of bias assessment | 11 | Specify the methods used to assess risk of bias in the included studies, including details of the tool(s) used, how many reviewers assessed each study and whether they worked independently, and if applicable, details of automation tools used in the process. | p. 2 |
| Effect measures | 12 | Specify for each outcome the effect measure(s) (e.g. risk ratio, mean difference) used in the synthesis or presentation of results. | p. 2 |
| Synthesis methods | 13a | Describe the processes used to decide which studies were eligible for each synthesis (e.g. tabulating the study intervention characteristics and comparing against the planned groups for each synthesis (item #5)). | N/A |
|  | 13b | Describe any methods required to prepare the data for presentation or synthesis, such as handling of missing summary statistics, or data conversions. | N/A |
|  | 13c | Describe any methods used to tabulate or visually display results of individual studies and syntheses. | p. 2 |
|  | 13d | Describe any methods used to synthesize results and provide a rationale for the choice(s). If meta-analysis was performed, describe the model(s), method(s) to identify the presence and extent of statistical heterogeneity, and software package(s) used. | p. 2 |
|  | 13e | Describe any methods used to explore possible causes of heterogeneity among study results (e.g. subgroup analysis, meta-regression). | p. 3 |
|  | 13f | Describe any sensitivity analyses conducted to assess robustness of the synthesized results. | p. 3 |
| Reporting bias assessment | 14 | Describe any methods used to assess risk of bias due to missing results in a synthesis (arising from reporting biases). | N/A |
| Certainty assessment | 15 | Describe any methods used to assess certainty (or confidence) in the body of evidence for an outcome. | N/A |
| **RESULTS** | | |  |
| Study selection | 16a | Describe the results of the search and selection process, from the number of records identified in the search to the number of studies included in the review, ideally using a flow diagram. | p. 3 / Figure 1 |
|  | 16b | Cite studies that might appear to meet the inclusion criteria, but which were excluded, and explain why they were excluded. | N/A |
| Study characteristics | 17 | Cite each included study and present its characteristics. | p. 3–4 / Table 1 |
| Risk of bias in studies | 18 | Present assessments of risk of bias for each included study. | p. 3 / Supplementary Table 3 |
| Results of individual studies | 19 | For all outcomes, present, for each study: (a) summary statistics for each group (where appropriate) and (b) an effect estimate and its precision (e.g. confidence/credible interval), ideally using structured tables or plots. | p. 4–5 / Figures 2–4 |
| Results of syntheses | 20a | For each synthesis, briefly summarise the characteristics and risk of bias among contributing studies. | p. 3–6 |
|  | 20b | Present results of all statistical syntheses conducted. If meta-analysis was done, present for each the summary estimate and its precision (e.g. confidence/credible interval) and measures of statistical heterogeneity. If comparing groups, describe the direction of the effect. | p. 5–6 |
|  | 20c | Present results of all investigations of possible causes of heterogeneity among study results. | p. 6 |
|  | 20d | Present results of all sensitivity analyses conducted to assess the robustness of the synthesized results. | p. 5 |
| Reporting biases | 21 | Present assessments of risk of bias due to missing results (arising from reporting biases) for each synthesis assessed. | N/A |
| Certainty of evidence | 22 | Present assessments of certainty (or confidence) in the body of evidence for each outcome assessed. | N/A |
| **DISCUSSION** | | |  |
| Discussion | 23a | Provide a general interpretation of the results in the context of other evidence. | p. 7–8 |
|  | 23b | Discuss any limitations of the evidence included in the review. | p. 8 |
|  | 23c | Discuss any limitations of the review processes used. | p. 8 |
|  | 23d | Discuss implications of the results for practice, policy, and future research. | p. 8 |
| **OTHER INFORMATION** | | |  |
| Registration and protocol | 24a | Provide registration information for the review, including register name and registration number, or state that the review was not registered. | p. 1–2 |
|  | 24b | Indicate where the review protocol can be accessed, or state that a protocol was not prepared. | N/A |
|  | 24c | Describe and explain any amendments to information provided at registration or in the protocol. | N/A |
| Support | 25 | Describe sources of financial or non-financial support for the review, and the role of the funders or sponsors in the review. | p. 9 |
| Competing interests | 26 | Declare any competing interests of review authors. | p. 9 |
| Availability of data, code and other materials | 27 | Report which of the following are publicly available and where they can be found: template data collection forms; data extracted from included studies; data used for all analyses; analytic code; any other materials used in the review. | p. 8 |

# **Supplementary Table 2**. Search strategies for Pubmed, Scopus, and Web of Science

| **Search Query** | **Search Fields** |
| --- | --- |
| **Pubmed** | |
| ("Non-alcoholic Fatty Liver Disease"[Mesh] OR "Fatty Liver"[Mesh] OR "Steatohepatitis, Nonalcoholic"[Mesh] OR NAFLD[tiab] OR "nonalcoholic fatty liver disease"[tiab] OR "non-alcoholic fatty liver disease"[tiab] OR MAFLD[tiab] OR "metabolic associated fatty liver disease"[tiab] OR "metabolic-associated fatty liver disease"[tiab] OR MASLD[tiab] OR "metabolic dysfunction-associated steatotic liver disease"[tiab] OR "metabolic dysfunction associated steatotic liver disease"[tiab] OR NASH[tiab] OR "nonalcoholic steatohepatitis"[tiab] OR "non-alcoholic steatohepatitis"[tiab] OR MASH[tiab] OR "metabolic dysfunction-associated steatohepatitis"[tiab] OR "metabolic dysfunction associated steatohepatitis"[tiab] OR "hepatic steatosis"[tiab] OR steatosis[tiab] OR "fatty liver"[tiab]) AND ("Atrial Fibrillation"[Mesh] OR "atrial fibrillation"[tiab] OR AF[tiab]) AND ( "Catheter Ablation"[Mesh] OR "Ablation Techniques"[Mesh] OR "Cryosurgery"[Mesh] OR ablation[tiab] OR "catheter ablation"[tiab] OR "radiofrequency ablation"[tiab] OR cryoablation[tiab] OR "pulmonary vein isolation"[tiab] OR PVI[tiab]]) | Title/Abstract |
| **Scopus** | |
| TITLE-ABS-KEY((NAFLD OR "nonalcoholic fatty liver disease" OR "non-alcoholic fatty liver disease" OR MAFLD OR "metabolic associated fatty liver disease" OR "metabolic-associated fatty liver disease" OR MASLD OR "metabolic dysfunction-associated steatotic liver disease" OR NASH OR "nonalcoholic steatohepatitis" OR "non-alcoholic steatohepatitis" OR MASH OR "metabolic dysfunction-associated steatohepatitis" OR "fatty liver" OR steatosis OR "hepatic steatosis") AND ("atrial fibrillation" OR AF) AND (ablation OR "catheter ablation" OR "pulmonary vein isolation" OR PVI OR cryoablation OR "radiofrequency ablation")) | Title/Abstract/Keyword plus/Author keywords |
| **Web of Science** | |
| (NAFLD OR "nonalcoholic fatty liver disease" OR "non-alcoholic fatty liver disease" OR MAFLD OR "metabolic associated fatty liver disease" OR "metabolic-associated fatty liver disease" OR MASLD OR "metabolic dysfunction-associated steatotic liver disease" OR NASH OR "nonalcoholic steatohepatitis" OR "non-alcoholic steatohepatitis" OR MASH OR "metabolic dysfunction-associated steatohepatitis" OR "fatty liver" OR steatosis OR "hepatic steatosis") AND ("atrial fibrillation" OR AF) AND (ablation OR "catheter ablation" OR "pulmonary vein isolation" OR PVI OR cryoablation OR "radiofrequency ablation") | Title/Abstract/Keywords |

# **Supplementary Table 3.** Risk of bias assessment.

| **Study** | **Selection** | **Comparability** | **Outcome** | **Overall** | **Risk of bias** |
| --- | --- | --- | --- | --- | --- |
| Takami et al. | 4/4 | 2/2 | 2/3 | 8/9 | Low |
| Donnellan et al. | 4/4 | 2/2 | 2/3 | 8/9 | Low |
| Wang et al. | 4/4 | 1/2 | 2/3 | 7/9 | Low |
| Wang et al. | 3/4 | 1/2 | 2/3 | 6/9 | Moderate |
| Wang et al. | 4/4 | 1/2 | 2/3 | 7/9 | Low |
| Decoin et al. | 3/4 | 1/2 | 2/3 | 6/9 | Moderate |
| Xu et al. | 4/4 | 1/2 | 2/3 | 7/9 | Low |

# **Supplementary Table 4.** Antiarrhythmic drug use in the included studies.

| **Study** | **Baseline AAD use** | **Follow-up AAD use** |
| --- | --- | --- |
| Takami et al. | MASLD: 29.9%, No MASLD: 36.1% | NR |
| Donnellan et al. | MASLD: 86.5%, No MASLD: 83.1% | Among patients who were taking AADs prior to ablation, these were continued for the first 3 months post-ablation. |
| Wang et al. | Overall: 84.3% | All patients were prescribed for 3 months post-ablation. |
| Wang et al. | NR | Usually prescribed during the initial 3-month blanking period, after which its discontinuation was strongly advised. |
| Wang et al. | Overall: 67.5% | All patients were prescribed for 3 months after ablation. Subsequent use was determined by the physicians and patients. |
| Decoin et al. | NR | NR |
| Xu et al. | Persistent AF patients received AADs under physician guidance unless contraindicated. Paroxysmal AF patients did not receive routine AADs. | All AADs were discontinued after the blanking period unless symptomatic atrial arrhythmias recurred. |

# **Supplementary Table 5.** Definitions of MASLD, AF recurrence and protocol for monitoring recurrence.

| **Study** | **MASLD definition** | **Recurrence** | **Recurrence monitoring** |
| --- | --- | --- | --- |
| Takami et al. | Based on CT or ultrasonography, while excluding cases of secondary hepatic fat accumulation | Any documented AF lasting >30 s detected using a 12-lead ECG or other appropriate test | Patients were followed up at 1, 3, 6, 12, and 24 months after the ablation procedure, which included physical examinations and 12-lead ECGs. Patients were instructed to self-monitor their pulse daily and to immediately report any pulse irregularity, which was followed by event monitoring or Holter monitoring. Furthermore, AF recurrence was assessed using implantable loop recorders or pacemakers/defibrillators, when available. |
| Donnellan et al. | 1) evidence of hepatic steatosis, either by imaging or histology; and 2) lack of secondary causes of hepatic fat accumulation | Recurrence was defined as documented atrial arrhythmia outside of the initial 3-month blanking period lasting 30 s or more and documented on 12-lead ECG, on Holter monitor, or during interrogation of implanted devices. | All patients wore transtelephonic rhythm transmitters for at least 3 months post-ablation, and weekly recordings of their cardiac rhythm was obtained in all cases. Additional event recorder monitoring beyond 3 months was obtained in patients who experienced recurrent symptoms or had documented arrhythmia during the initial 3-month period. All patients were followed for at least 12 months post-ablation, and had scheduled clinic visits at 3, 6, and 12 months post– pulmonary vein isolation and annually thereafter. Recurrence data were obtained from transtelephonic monitoring, intracardiac cardioverter-defibrillator and permanent pacemaker interrogation where applicable, extended outpatient cardiac monitoring devices, electrocardiography (ECG) analysis, and patient-reported symptoms at follow-up clinic visits. |
| Wang et al. | 1) ultrasound imaging of the liver revealing diffuse fatty infiltration, 2) the exclusion of other conditions that may lead to fatty liver disease, such as autoimmune liver diseases, drug-induced liver injury, and viral hepatitis, and 3) an absence of excessive alcohol consumption | AF recurrence was defined as any atrial tachyarrhythmia lasting longer than 30 seconds as detected by electrocardiogram or Holter monitoring, after the initial 3-month blanking period. | All patient follow-up evaluations were conducted every three months during the first year after the procedure, either in outpatient settings or via telephone. (Some patients received outpatient treatment at local hospitals and were followed up by personnel via phone.) Each follow-up included a 12-lead electrocardiogram, 24-hour Holter monitoring, and clinical evaluation. Patients with AF-related symptoms were asked to schedule an extra outpatient visit. |
| Wang et al. | MASLD was defined as the presence of hepatic steatosis (FLI >=60) along with ≥ 1 pre-defined cardiometabolic risk factor, such as overweight or abnormalities in glucose, blood pressure, lipids, or high-density lipoprotein cholesterol, excluding secondary liver steatosis causes | AF recurrence was defined as any AF, atrial flutter (AFL) or atrial tachycardia (AT) episode lasting at least 30 s, irrespective of AAD use. Clinical recurrence was identified as any electrocardiographic evidence of AF after the 3-month blanking period. | follow-up visits at our outpatient clinic at 3-, 6-, and 12-month post-ablation, and then every 12 months or whenever symptoms reappeared. Rhythm monitoring during these visits included clinical assessments for AF recurrence, routine electrocardiograms, and Holter monitoring. In cases where patients experienced palpitations, an ECG or Holter was performed to detect any arrhythmic recurrence. |
| Wang et al. | non-excessive alcohol consumption, detection of hepatic steatosis by ultrasound, and appropriate exclusion  of other liver diseases | Recurrent AF was defined as any atrial arrhythmia lasting for more than 30 s based on a 12-lead electrocardiogram or 24 h Holter monitoring after the 3-month blanking period | Patients were scheduled for follow-up in the outpatient setting at 3-month intervals during the first year after RFCA. Patients who had any symptoms related to AF were asked to immediately complete an additional outpatient visit. |
| Decoin et al. | FLI >=60 and metabolic syndrome | documented AF or atrial flutter on 12-lead electrocardiogram and/or episode >30 s during Holter monitoring. | Within the first year after ablation, our inhouse protocol unfolds electrocardiograms when symptoms are reported, during outpatient visits in our tertiary hospital, or in the treating cardiologist’s office (at 3, 6, and 12 months). Holter monitoring at 24 to 48 h was also performed at 3 and 6 months. The treating cardiologists then followed up the patients, with the number of outpatient visits at clinician discretion. A 90-day "blanking period” was followed. |
| Xu et al. | Diagnostic criteria established by the AASLD (imaging/biopsy + exclusion of secondary causes) All enrolled patients with hepatic steatosis would also meet the MASLD criteria. | ECG-documented atrial tachyarrhythmia (AF/atrial flutter/tachycardia) lasting >30 s beyond the 3-month blanking period. | All patients completed ≥12 months of follow-up after ablation, and had scheduled clinic visits at 3, 6, and 12 months. Patients who had any symptoms related to AF were asked to immediately complete an additional outpatient visit. Recurrence data were obtained from ECG analysis, and 3-day Holter monitoring. |

# **Supplementary Table 6.** Additional population characteristics of the included studies.

|  | **Age (MASLD)** | **Age (No MASLD)** | **Male (MASLD)** | **Male (No MASLD)** | **DM (MASLD)** | **DM (No MASLD)** | **HTN (MASLD)** | **HTN (No MASLD)** | **BMI (MASLD)** | **BMI (No MASLD)** | **PeAF (MASLD)** | **PeAF (No MASLD)** |
| --- | --- | --- | --- | --- | --- | --- | --- | --- | --- | --- | --- | --- |
| Takami et al. | 63 | 68 | 71.1 | 71.8 | 35.1 | 17.2 | 59.7 | 52.7 | 26 | 23 | 38.3 | 34 |
| Donnellan et al. | 65.4 | 65.6 | 69 | 69 | 25 | 14 | 74 | 71 | 33 | 32.5 | 58 | 56 |
| Wang et al. | NR | NR | NR | NR | NR | NR | NR | NR | NR | NR | NR | NR |
| Wang et al. | 57.7 | 61.8 | 76.1 | 59.1 | 11.3 | 12.1 | 53.5 | 45.3 | 27.9 | 24.1 | 39.4 | 38.8 |
| Wang et al. | NR | NR | NR | NR | NR | NR | NR | NR | NR | NR | NR | NR |
| Decoin et al. | 58.1 | 60.3 | 77 | 54 | 22 | 12 | 55 | 29 | 31.4 | 24.7 | 56 | 41 |
| Xu et al. | 61.1 | 63 | 62.3 | 67.9 | 18.9 | 11.3 | 43.4 | 32.1 | 26 | 24.5 | 30.2 | 41.5 |
| DM: diabetes mellitus, HTN: hypertension, BMI: body mass index, PeAF: persistent AF | | | | | | | | | | | | |

# **Supplementary Table 7.** Confounders adjusted for in multivariate models of the association between MASLD and AF recurrence after CA.

| **Study** | **Confounders** |
| --- | --- |
| Takami et al. | Age, female sex, body mass index, heart failure, diabetes mellitus, paroxysmal AF, BNP, HbA1c, HOMA-IR, ALT, AST, PLT count, albumin, CRP, L/S ratio, FIB-4, LAVi, RF ablation |
| Donnellan et al. | LAVi >= URL, HbA1c >= 7%, body mass index >= 31.7kg/m^2^, statin use, AST, Nonparoxysmal AF, obstructive sleep apnea syndrome, heart failure, NTproBNP> 325 |
| Wang et al. | Age, sex, body mass index, current drinking, diabetes mellitus, AF duration (≥24 months), paroxysmal AF, LA diameter, triglycerides, and CHA_2_DS_2_-VASc score |
| Wang et al. | Age, sex, AF duration, AF type, alcohol, hemoglobin, LA diameter, LVEF |
| Xu et al. | Persistent AF, LA diameter, LVMi, NTproBNP >300 |
| AF: atrial fibrillation, BNP: brain natriuretic peptide, HOMA-IR: Homeostasis Model Assessment - Insulin Resistance, ALT: alaline aminotransferase, AST: aspartate aminotransferase, PLT: platelet, CRP: C-reactive protein, LAVi: left atrial volume index, RF: radiofrequency, URL: upper reference limit, LVEF: left ventricular ejection fraction | |

# **Supplementary Fig. 1.** Leave-one-out sensitivity analysis of the MASLD meta-analysis.

# **Supplementary Fig. 2.** Subgroup analysis of MASLD and AF recurrence after CA.

# **Supplementary Fig. 3.** Contour-enhanced funnel plot assessing publication bias for the MASLD meta-analysis.

# **Supplementary Fig. 4.** Contour-enhanced funnel plot assessing publication bias for the FIB4 meta-analysis.

# **Supplementary Fig. 5.** Contour-enhanced funnel plot assessing publication bias for the NFS meta-analysis.
